# A CD22-reactive TCR from the T-cell allorepertoire for the treatment of acute lymphoblastic leukemia by TCR gene transfer

**DOI:** 10.18632/oncotarget.12247

**Published:** 2016-09-26

**Authors:** Lorenz Jahn, Renate S. Hagedoorn, Dirk M. van der Steen, Pleun Hombrink, Michel G.D. Kester, Marjolein P. Schoonakker, Daniëlle de Ridder, Peter A. van Veelen, J.H. Frederik Falkenburg, Mirjam H.M. Heemskerk

**Affiliations:** ^1^ Department of Hematology, Leiden University Medical Center, 2300 RC Leiden, The Netherlands; ^2^ Department of Hematopoiesis, Sanquin Research, 1006 AD Amsterdam, The Netherlands; ^3^ Center for Proteomics and Metabolomics, Leiden University Medical Center, 2300 RC Leiden, The Netherlands

**Keywords:** CD22, TCR gene transfer, immunotherapy, acute lymphoblastic leukemia, allogeneic HLA

## Abstract

CD22 is currently evaluated as a target-antigen for the treatment of B-cell malignancies using chimeric antigen receptor (CAR)-engineered T-cells or monoclonal antibodies (mAbs). CAR- and mAbs-based immunotherapies have been successfully applied targeting other antigens, however, occurrence of refractory disease to these interventions urges the identification of additional strategies. Here, we identified a TCR recognizing the CD22-derived peptide RPFPPHIQL (CD22_RPF_) presented in human leukocyte antigen (HLA)-B^*^07:02. To overcome tolerance to self-antigens such as CD22, we exploited the immunogenicity of allogeneic HLA. CD22_RPF_-specific T-cell clone 9D4 was isolated from a healthy HLA-B^*^07:02^neg^ individual, efficiently produced cytokines upon stimulation with primary acute lymphoblastic leukemia and healthy B-cells, but did not react towards healthy hematopoietic and nonhematopoietic cell subsets, including dendritic cells (DCs) and macrophages expressing low levels of CD22. Gene transfer of TCR-9D4 installed potent CD22-specificity onto recipient CD8+ T-cells that recognized and lysed primary B-cell leukemia. TCR-transduced T-cells spared healthy CD22^neg^ hematopoietic cell subsets but weakly lysed CD22^low^-expressing DCs and macrophages. CD22-specific TCR-engineered T-cells could form an additional immunotherapeutic strategy with a complementary role to CAR- and antibody-based interventions in the treatment of B-cell malignancies. However, CD22 expression on non-B-cells may limit the attractiveness of CD22 as target-antigen in cellular immunotherapy.

## INTRODUCTION

Immunotherapy for the treatment of hematological malignancies of the B-cell lineage using monoclonal antibodies (mAb) or chimeric antigen receptor (CAR)-modified T-cells has demonstrated clinical efficacy. Complete remissions have been achieved in chronic lymphocytic leukemia (CLL) and acute lymphoblastic leukemia (ALL) by CD19-targeting CAR T-cells [1–4]. CD20-specific mAb such as rituximab have been successfully applied in the treatment of B-cell malignancies [5–8]. Due to the high expression of CD19 and CD20 on malignant cells, these antigens can be efficiently targeted. Although healthy B-cells also express CD19 and CD20, and are depleted in the course of CAR or mAb treatment, long-term B-cell aplasia is tolerable and well manageable from a clinical perspective [4, 9–11]. However, emergence of antigen-loss tumor-escape variants after administration of such interventions can pose a serious problem. Grupp *et al.* have reported on 13 out of 53 patients suffering from the relapse of CD19^neg^ ALL after treatment with CD19-specific CAR T cells [12]. Although 76% of patient suffering from diffuse large B-cell lymphoma benefited from the addition of rituximab to standard chemotherapy [5], the overall survival is significantly worse in patients in need of second administration of rituximab treatment compared to rituximab-naïve patients (37% vs 67%) [13]. These outcomes highlight the importance to identify additional antigenic targets.

CD22 belongs to the sialic acid binding Ig-like lectin (Siglec) family [14]. CD22 is expressed at low levels on B-cell progenitors and strongly on mature B-cells. Via its extracellular domain CD22 binds to sialylated carbohydrates, while its intracellular domain contains immunoreceptor tyrosine-based inhibitory motifs (ITIMs) capable of activating phosphatases which in turn can dampen positive components of the B-cell receptor signaling cascade [15]. Therefore, CD22 acts predominately as an inhibitory coreceptor and plays an important role in BCR signaling threshold.

Due to the expression of CD22 not only on healthy but also malignant B-cells, CD22 is currently assessed as a target for CAR- and mAb-based immunotherapy approaches in the treatment of mainly ALL and B-cell lymphoma [16–19].

Besides CAR- and mAb-based strategies, the administration of T-cell receptor (TCR)-modified T-cells has emerged as a promising intervention of solid tumors [20, 21]. TCRs induce T-cell activation by binding to cognate antigen-derived peptides presented on the cell surface in the context of major histocompatibility complex (MHC). Since MHC molecules sample the cell's proteome, TCRs can target peptides derived from intra- and extracellular proteins. Hence, TCRs can still efficiently target antigens whose extracellular abundance may be insufficient to be susceptible to CAR- or mAb-based immunotherapies. Therefore, TCR-modified T-cells form an additional avenue in the exploitation of promising antigenic targets.

Here, we describe the identification of a TCR specifically recognizing the CD22-derived peptide CD22_RPF_ presented in the context of the human leukocyte antigen (HLA)-B*07:02. To effectively target self-antigens such as CD22, we exploited the immunogenicity of allogeneic (non-self) HLA (alloHLA). From an HLA-B7^neg^ healthy individual we isolated T-cell clone 9D4 that expressed a CD22_RPF_-specific TCR. Clone 9D4 recognized HLA-B7^pos^ primary ALL samples, ALL cell lines and healthy B-cells. Using TCR gene transfer, TCR-9D4 modified CD8^+^ T-cells recognized and lysed primary ALL samples, ALL cell lines, and healthy B-cells. TCR-transduced T-cells did not produce cytokine upon stimulation with but weakly lysed dendritic cells (DCs) and macrophages expressing low levels of CD22. CD22-specific TCR-engineered T-cells could form an additional strategy to exploit CD22 as antigenic target in immunotherapy of B-cell malignancies. However, due to the expression of CD22 on non-B-cells, our data also indicate potential limitations of CD22 as a target-antigen in cell-based immunotherapeutic strategies.

## RESULTS

### Identification of a CD22 epitope

From the HLA-ligandome of B lymphocytes [22], we identified a CD22-derived nonameric peptide RPFPPHIQL (CD22_RPF_), that is naturally processed and presented in the context of HLA class I. Matching mass spectrometry fragmentation patterns of synthesized and eluted peptide indicated correct identification (Supplementary Figure S1). Using a public prediction algorithm [23, 24], peptide CD22_RPF_ was designated to be a strong binder for HLA-B*07:02 (HLA-B7). This prediction was supported by the formation of stable peptide-MHC (pMHC) monomers composed of CD22_RPF_ and HLA-B7.

### Isolation of CD22-reactive T-cell clone 9D4

**Figure 1:**
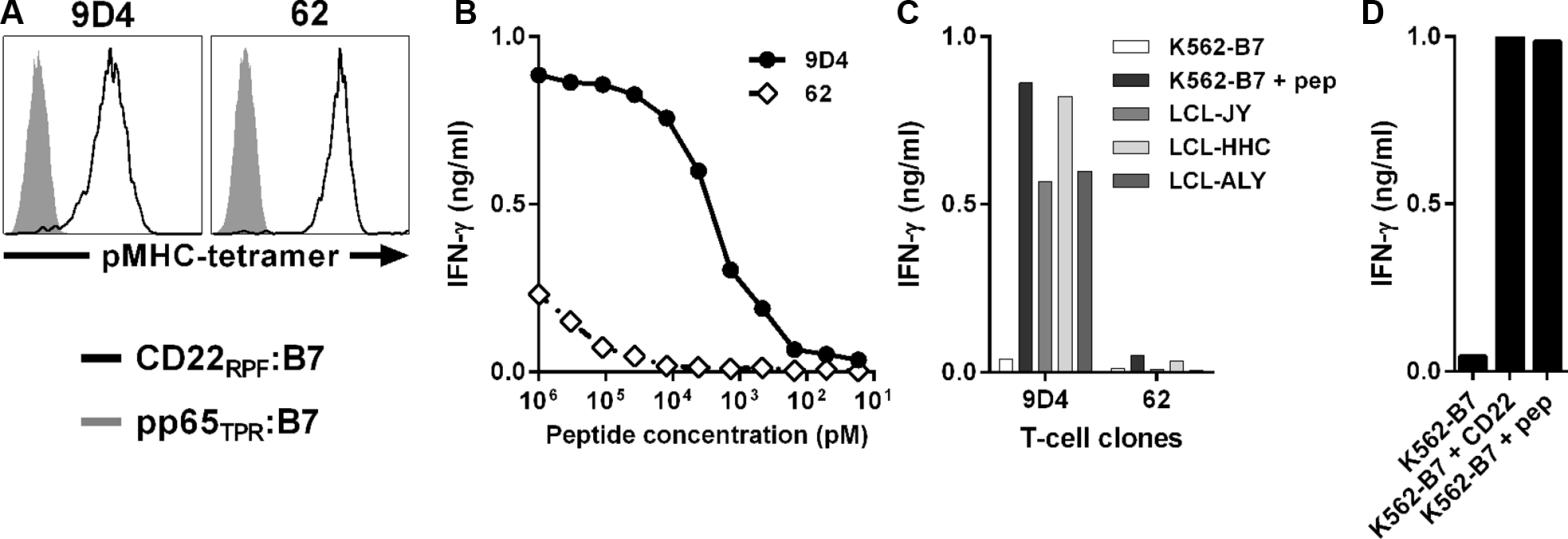
Identification of CD22-reactive T-cell clone 9D4 from an HLA-B7^neg^ healthy individual T-cell clones were isolated from HLA-B7^neg^ healthy individuals by pMHC-tetramers composed of CD22_RPF_ bound to HLA-B7 (CD22_RPF_:B7) (black), and selected for CD22 specificity. (**A**) Shown are histograms of two representative T-cell clones stained with specific pMHC-tetramer CD22_RPF_:B7 or a control pMHC-tetramer composed of pp65-derived peptide TPRVTGGGAM bound to HLA-B7 (pp65_TPR_:B7) (gray). (**B**–**C**) T-cell clones 9D4 and 62 were co-cultured with CD22^neg^ K562-B7 cells exogenously loaded with peptide CD22_RPF_ (B) or three HLA-B7^pos^ B-LCLs naturally expressing CD22 (C) at 1:15 responder-to-stimulator ratio. (**D**) T-cell clone 9D4 was incubated with K562-B7 cells stably transduced to express CD22 (K562-B7 + CD22). K562-B7 loaded with peptide CD22_RPF_ (K562-B7 + pep) served as positive control. Shown are means of one representative experiment carried out in duplicate.

To isolate CD22-reactive T-cell clones, we used pMHC-tetramers composed of CD22_RPF_ bound HLA-B7. Starting with 250 to 1,000×10^6^ peripheral blood mononuclear cells (PBMCs) from six healthy HLA-B7^neg^ individuals, pMHC-tetramer-binding cells were first enriched by magnetic activated cell sorting (MACS). From the positive cell fraction, containing pMHC-tetramer binding cells, hundreds of pMHC-tetramer^+^ CD8^+^ T-cells were then acquired by single-cell FACSorting and clonally expanded for two weeks. From the expanding clones, 7 CD22-reactive T-cell clones were identified based on their reactivity towards K562 cells expressing HLA-B7 (K562-B7) exogenously loaded with CD22_RPF_ in the absence of reactivity towards unloaded K562-B7 cells. Among these 7 T-cell clones, clone 9D4 demonstrated highest peptide sensitivity when stimulated with titrated amounts of CD22_RPF_ and robust recognition of three HLA-B7^pos^ CD22-expressing Epstein-Barr virus (EBV)-transformed B-lymphoblastic cell lines (B-LCLs) (Figure 1A–1C). In contrast, other clones, as exemplified by clone 62, required high amounts of exogenous peptide to be stimulated and failed to recognize endogenously processed antigen (Figure 1A–1C).

Reactivity towards stimulator cells by clone 9D4 was CD22 dependent as introduction of CD22 into otherwise CD22-negative K562-B7 resulted in their recognition (Figure 1D).

In summary, high avidity CD22-reactive T-cell clone 9D4 was isolated from an HLA-B7^neg^ healthy individual by using pMHC-tetramers composed of peptide CD22_RPF_ bound to HLA-B7. Clone 9D4 recognized endogenously processed and presented peptide CD22_RPF_.

### Recognition of B-cell malignancies by clone 9D4

**Figure 2:**
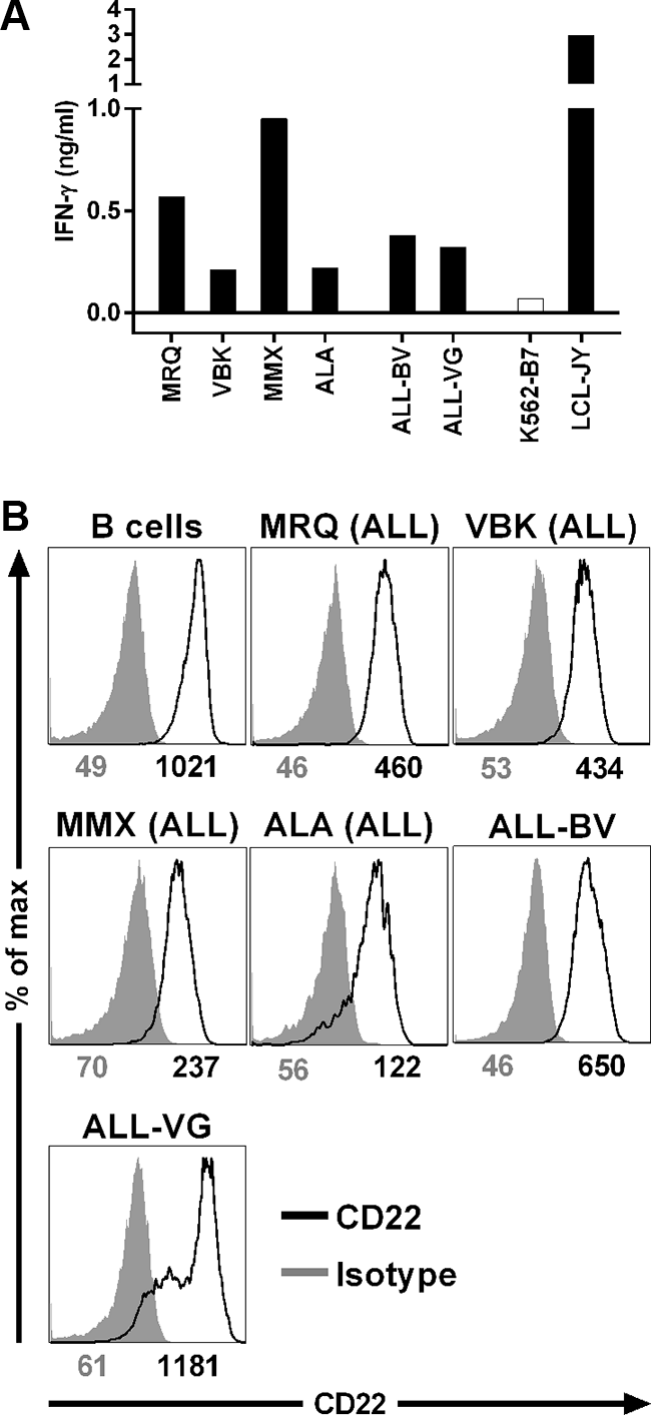
Recognition of B-cell malignancies by T-cell clone 9D4 (**A**) CD22-specific T-cell clone 9D4 was cocultured with primary HLA-B7^pos^ acute lymphoblastic leukemia (ALL; MRQ, VBK, MMX, and ALA) or two ALL cell lines (ALL-BV and ALL-VG) at a 1:25 responder-to-stimulator ratio. CD22^neg^ K562-B7 cells and CD22^pos^ LCL-JY served as negative and positive control, respectively. (**B**) Shown are histograms of primary malignant B-cell samples and ALL cell lines stained with a CD22-specific antibody (black) or an isotype control (gray). Samples include primary ALL and ALL cell lines used to stimulate clone 9D4 in (A). Healthy B-cells served as positive control. Numbers under histograms indicate mean fluorescent intensity of respective peak.

We tested the capacity of clone 9D4 to recognize HLA-B7^pos^ B-cell malignancies. Clone 9D4 was stimulated with samples from patients at diagnosis suffering from acute lymphoblastic leukemia (ALL) and ALL cell lines (Figure 2A). Clone 9D4 recognized all 4 primary ALL samples and both ALL cell lines with varying degrees of cytokine secretion in response to each sample. FACS analysis demonstrated that cell surface expression of CD22 varied between samples (Figure 2B). Normal B-cells and ALL cell line ALL-VG demonstrated highest CD22 expression followed by ALL-BV. CD22 was expressed to lower degrees on primary ALL samples. CD22 cell surface expression correlated with CD22 mRNA expression (data not shown). However, although ALL samples MMX had lower CD22 expression compared to sample MRQ, both samples were equally well recognized, indicating that also reduced levels of CD22 can be detected by this T-cell clone.

These data indicated that CD22-reactive T-cell clone 9D4 can recognize CD22-expressing primary HLA-B7^pos^ B-cell malignancies such as ALL.

### B-cell-restricted recognition profile of clone 9D4

**Figure 3:**
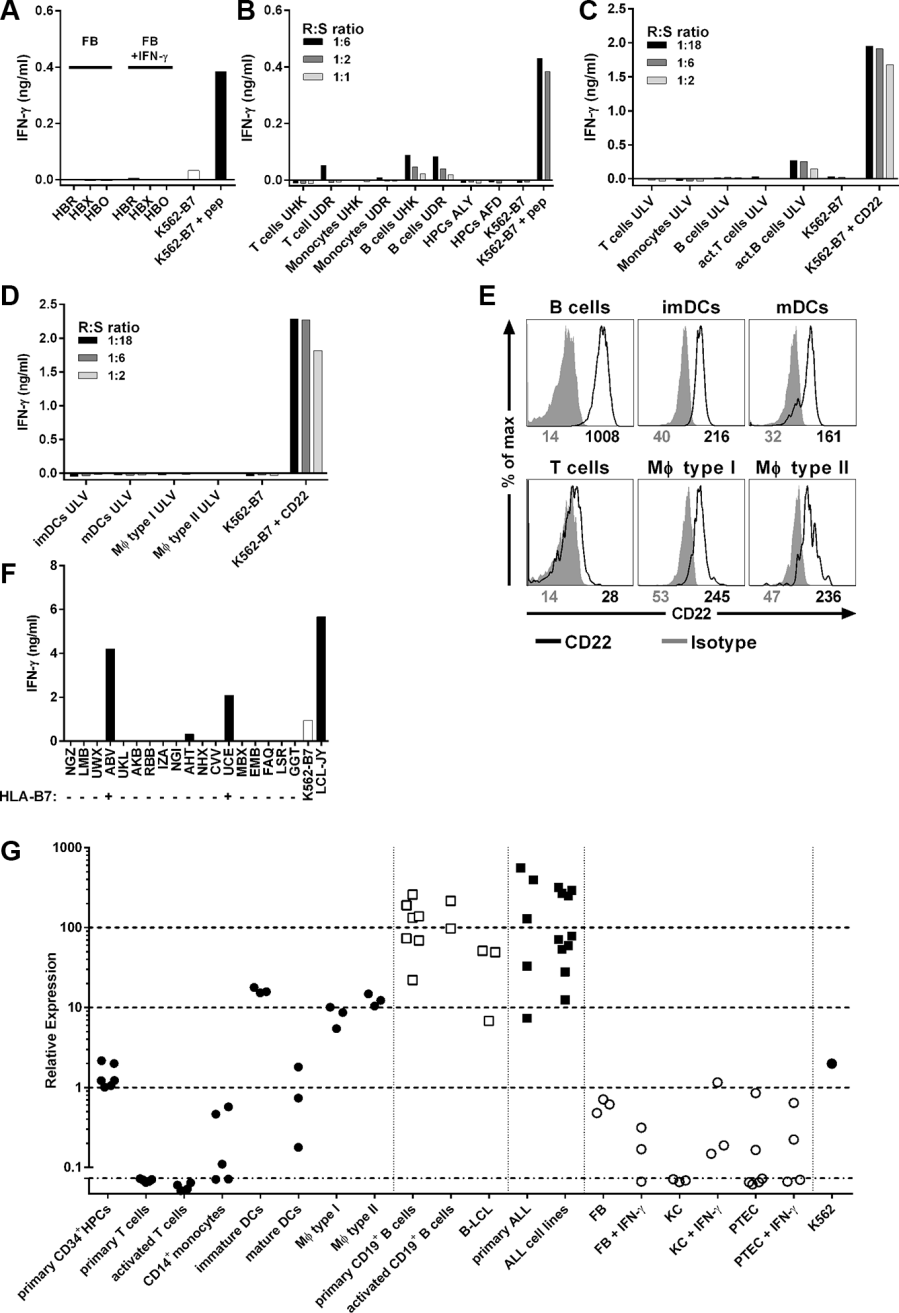
B-cell-restricted reactivity profile of T-cell clone 9D4 (**A**) Clone 9D4 was coincubated with HLA-B7^pos^ Fibroblast (FB) from three healthy individuals (HBR, HBX, HBO) at a 1:6 responder-to-stimulator ratio. Fibroblasts were treated with IFN-γ (FB + IFN-γ) for 4 days prior to experiment to simulate inflamed conditions. CD22^neg^ K562-B7 served as negative control. K562-B7 pulsed with CD22_RPF_ (+ pep) served as positive control. (**B**–**D**) Healthy hematopoietic cell subsets from 5 different HLA-B7^pos^ individuals (UHK, UDR, ALY, AFD, ULV) were coincubated with clone 9D4 at various responder-to-stimulator (R:S) ratios. Primary T cells, monocytes, B cells, and hematopoietic progenitor cells (HPCs) were isolated from cryopreserved material (B). T cells were stimulated with PHA (act. T cells). B cells were stimulated with CD40L (act. B cells) (C). Immature and mature dendritic cells (imDCs and mDCs, respectively), and type I and II macrophages (MΦ type I and type II, respectively) were monocyte-derived (D). CD22^neg^ K562-B7 served as negative control. K562-B7 pulsed with CD22_RPF_ (+ pep) or expressing CD22 (+ CD22) served as positive control. (**E**) Shown are histograms of various healthy hematopoietic subsets stained with a CD22-specific antibody (black) or an isotype control (gray). Numbers under histograms indicate mean fluorescent intensity of respective peak. (**F**) Clone 9D4 was cocultured with HLA-B7^pos^ B-LCLs (+) or HLA-B7^neg^ B-LCLs (−) as indicated on the x-axis at a 1:15 responder-to-stimulator ratio. CD22^neg^ K562-B7 and LCL-JY served as negative and positive control, respectively. A complete list of all HLA class I and II molecules present can be found in Supplementary Table S1. (**G**) CD22 mRNA expression was measured in various hematopoietic and non-hematopoietic cell subsets by quantitative real-time PCR. Shown are samples of healthy hematopoietic origin (black dots), healthy B-cells (clear squares), B-cell malignancies (black squares) and samples of non-hematopoietic origin (clear dots). Individual dots indicate individual samples. Average expression in healthy B-cells was set to 100. Fibroblasts (FB), keratinocytes (KC) and proximal tubular epithelial cells (PTEC) were cocultured in the presence of IFN-γ (+ IFN-γ) for 4 days to simulate inflammation.

To investigate potential on- and off-target toxicity, T-cell clone 9D4 was stimulated with a panel of HLA-B7^pos^ healthy hematopoietic and nonhematopoietic cell subsets. No recognition of 3 fibroblasts was observed (Figure 3A). Moreover, clone 9D4 did not react towards fibroblasts that had been cultured in the presence of IFN-γ to simulate inflamed conditions.

When cocultured with primary hematopoietic cells of three healthy HLA-B7^pos^ individuals, T-cell clone 9D4 did not react towards T-cells, CD14^+^ monocytes or CD34^+^ hematopoietic precursor cells (HPCs) (Figure 3B–3C). On the other hand, clone 9D4 weakly recognized CD22-expressing primary and activated B-cells. Furthermore, no IFN-γ production of clone 9D4 was observed towards monocyte-derived immature and mature dendritic cells (DCs), and type I and II macrophages although low levels of mRNA and cell surface-expressed CD22 was detected in these cell samples (Figure 3D–3E and 3G). Clone 9D4 did not produce any GM-CSF upon stimulation with DCs or macrophages (data not shown). A control T-cell clone specific for a ubiquitously expressed antigen verified the stimulatory capacity of all tested cell samples (Supplementary Figure S2).

Finally, clone 9D4 showed no reactivity with other HLA class I molecules other than HLA-B7 when stimulated with a panel of B-LCLs expressing ca. 95% of common and rare HLA class I alleles [25] (Figure 3F and Supplementary Table S1).

In summary, these data indicated a safe reactivity profile of clone 9D4; CD22-expressing healthy B-cells were weakly recognized whereas no reactivity towards nonhematopoietic and hematopoietic cells not belonging to the B-cell compartment was observerd. Furthermore, clone 9D4 demonstrated a strong HLA-B7 dependency with no reactivity towards other tested HLA class I alleles.

### CD22 reactivity of TCR-modified T-cells by TCR gene transfer

**Figure 4:**
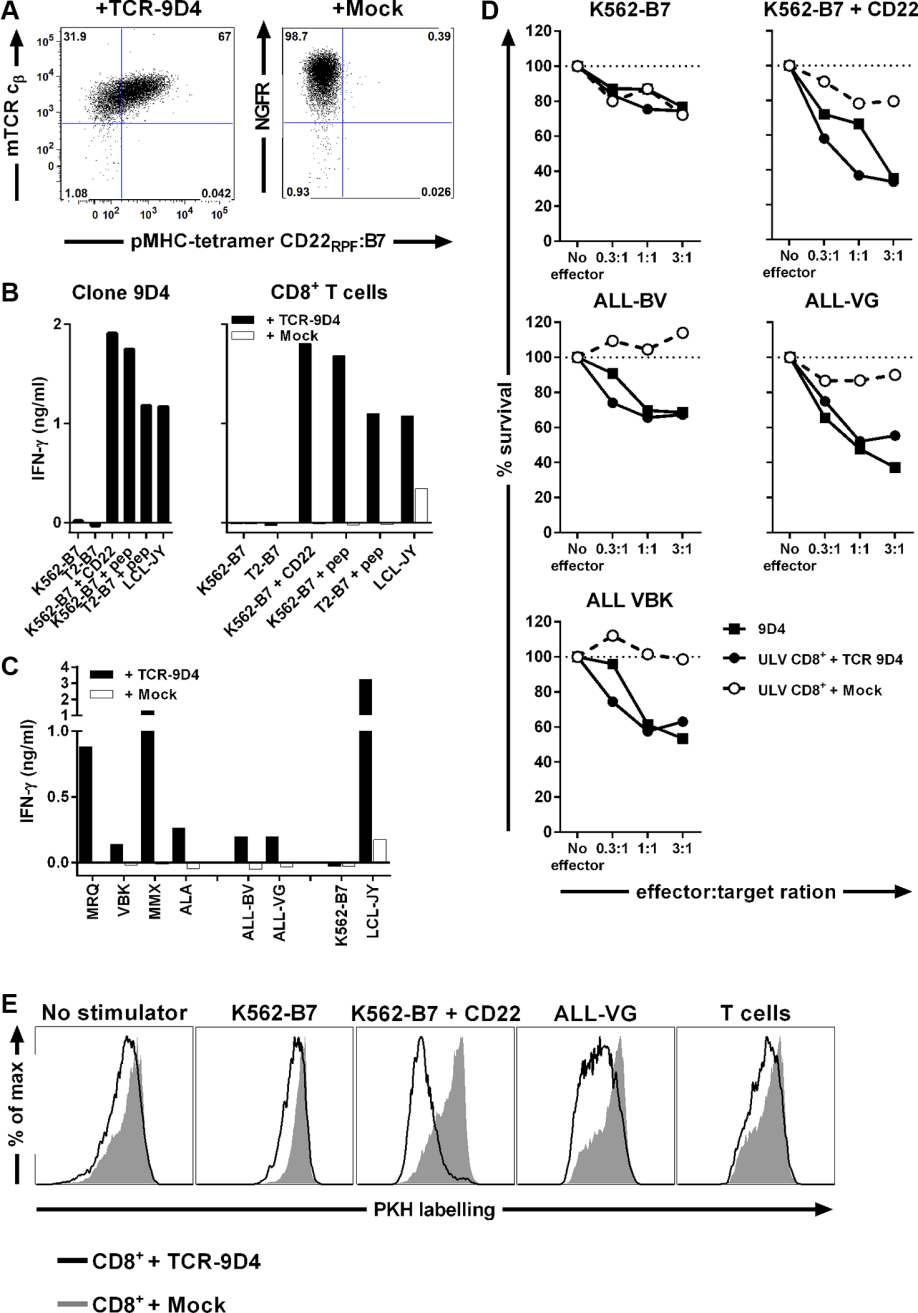
TCR gene transfer of TCR-9D4 installs CD22-specific reactivity onto recipient CD8^+^ T-cells leading to lysis of B-cell malignancies Retroviral transduction was used to introduce the TCR of clone 9D4 (TCR-9D4) in recipient CD8^+^ T-cells of an HLA-B7^pos^ healthy individual. An empty expression vector containing NGF-R as marker gene served as control (+ Mock) (**A**) Shown are dot plots of purified CD8^+^ T-cells transduced with TCR-9D4 or mock-transduced stained with pMHC-tetramer CD22_RPF_:B7. Transduction is monitored with an antibody binding to the murine TCR beta constant domain (mTCR c_β_) of TCR-9D4 or a NGF-R-specific antibody. (**B**) Original clone 9D4, purified TCR- or mock-transduced CD8^+^ T-cells were stimulated with CD22^neg^ K562-B7 cells or T2 cells expressing HLA-B7 (T2-B7), K562-B7 cells expressing CD22 (+ CD22), K562-B7 and T2-B7 cells pulsed with exogenous peptide CD22_RPF_ (+ pep), and CD22-expressing LCL-JY at a 1:15 responder-to-stimulator ratio. (**C**) Transduced T-cells were stimulated with HLA-B7^pos^ primary acute lymphoblastic leukemia MRQ, VBK, MMX, and ALA or two ALL cell lines (ALL-BV and ALL-VG). CD22^neg^ K562-B7 cells and CD22-expressing LCL-JY served as negative and positive control, respectively. (**D**) Original clone 9D4, TCR- or mock-transduced T-cells were cocultured with different HLA-B7^pos^ target cells at various effector-to-target ratios. Targets include two ALL cell lines (ALL-BV and ALL-VG) and primary ALL sample (VBK). Surival of target cells was assessed after 20 hours of coincubation. Shown are means of one experiment carried out in triplicate. (**E**) PKH-labelled TCR- or mock-transduced T-cells were cocultured with various irradiated stimulator cells for 5 days at 1:5 responder-to-stimulator ratio.

We sequenced the TCR of clone 9D4 (TCR-9D4) and constructed a retroviral vector which allows for the transduction of recipient T-cells. To enhance cell surface expression and preferential pairing of the introduced TCR alpha and beta chain, the V(D)J regions of TCR-9D4 were codon-optimized and fused to murine TCR constant domains. Retroviral transduction of recipient CD8^+^ T-cells led to cell surface expression of TCR-9D4 measured by staining of the murine TCR beta constant domain and binding to pMHC-tetramer CD22_RPF_:B7 (Figure 4A). TCR-transduced CD8^+^ T-cells highly recognized CD22-expressing stimulator cells comparable to the parental clone 9D4 (Figure 4B). The recognition was specific since mock-transduced T-cells failed to produce any cytokine upon stimulation, except for some background cytokine secretion against EBV-transformed LCL-JY most likely caused by the presence of EBV-reactive T-cells. When stimulated with a panel of primary B-cell malignancies, TCR-transduced but not mock-transduced T-cells produced IFN-γ upon coincubation (Figure 4C). TCR-engineered T-cells reacted towards all 4 ALL samples and both tested ALL cell lines with different amounts of IFN-γ production. Furthermore, CD22-specific TCR-transduced T-cells and parental clone 9D4 lysed primary ALL and ALL cell lines (Figure 4D). Lysis of primary ALL and ALL cell lines was comparable although CD22 expression differed between samples as shown previously in Figure 2B. Cell lysis was specific as CD22^neg^ K562-B7 cells were not recognized by either TCR-modified T-cells or the parental clone. Furthermore, mock-transduced T-cells did not lyse any sample indicating that reactivity towards target cells was introduced with TCR gene transfer. In addition, TCR-modified T-cells proliferated when cocultured with a CD22-expressing stimulator cell line K562-B7 + CD22 and ALL-VG but did not expand in the presence of CD22^neg^ stimulators such as autologous T-cells or K562-B7 (Figure 4E).

**Figure 5:**
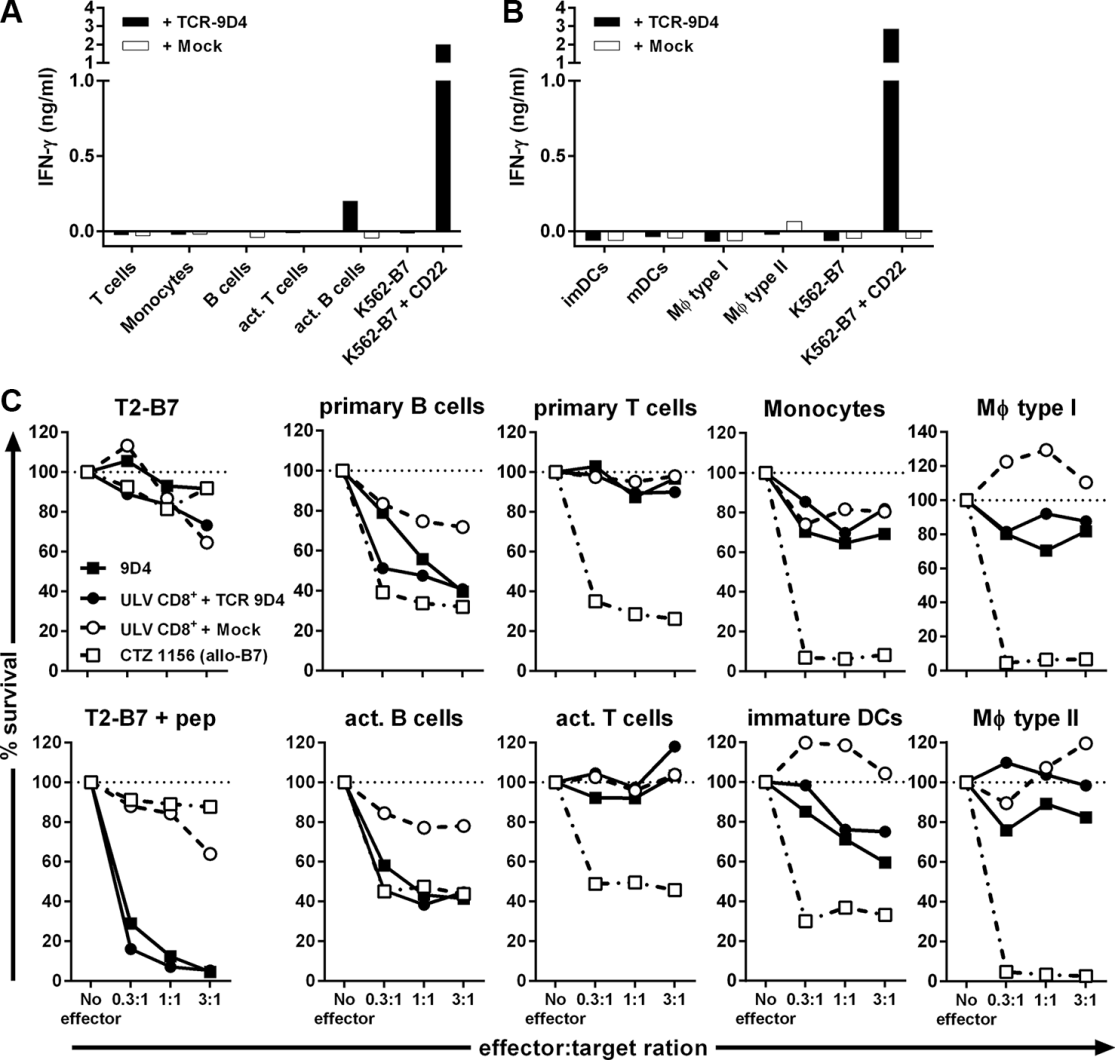
Off-tumor on-target toxicity of CD22-specific T cells caused by CD22 expression on dendritic cells and macrophages CD8^+^ T-cells of an HLA-B7^pos^ individual were retrovirally transduced to express either TCR-9D4 (+ TCR-9D4) or an empty vector (+ Mock). (**A**–**B**) Purified TCR- or mock-transduced CD8^+^ T-cells were stimulated with hematopoietic cell subsets of autologous origin. (**C**) Original clone 9D4, purified TCR- or mock-transduced CD8^+^ T-cells, or control clone CTZ were cocultured with T2-B7 cells or T2-B7 cells pulsed with peptide CD22_RPF_ (+ pep), or hematopoietic cell subsets derived from the same origin as TCR- and mock-transduced T-cells (autologous setting). Survival of target cells was assessed after 20 hours of coculture. Shown are means of one experiment carried out in triplicate.

When stimulated with healthy hematopoietic cell subsets of autologous origin, TCR-transduced T-cells did not react towards primary B-cells but recognized activated B-cells (Figure 5A). In contrast, TCR-engineered T-cells did not produce IFN-γ upon stimulation with primary or activated T-cells, primary CD14^+^ monocytes or monocyte-derived immature and mature DCs, and type I and II macrophages (Figure 5B). When tested for their lytic capacity, TCR-modified T-cells lysed autologous primary and activated B-cells (Figure 5C). Although weak lytic activity against immature DCs and type I macrophages was seen, no lysis of primary or activated T-cells, CD14^+^ monocytes or monocyte-derived type II macrophages was observed whereas a control T-cell clone lysed all target cells.

In summary, gene transfer of TCR-9D4 installed CD22-specific reactivity onto CD8^+^ T-cells. TCR-transduced CD8^+^ T-cells recognized and lysed primary B-cell malignancies, healthy B-cells and to a lesser degree also immature DCs and type I macrophages. No reactivity towards other CD22^neg^ healthy hematopoietic cell subsets outside of the B-cell compartment was observed.

## DISCUSSION

Here, we describe the identification of a CD22-specific TCR directed against the CD22-derived peptide RPFPPHIQL presented in the context of HLA-B7. TCR gene transfer installed CD22 reactivity onto recipient T-cells which recognized and lysed CD22-expressing primary ALL samples, ALL cell lines, and B-cells, while sparing healthy nonhematopoietic and hematopoietic cell subsets. However, although clone 9D4 or TCR-modified T-cells did not produce IFN-γ upon stimulation with DCs or macrophages, these cell subsets were weakly lysed and demonstrated low levels of CD22 expression.

This high affinity CD22-specific TCR was identified from T-cell clone 9D4 which was isolated from an HLA-B7^neg^ healthy individual. By exploiting the immunogenicity of allogeneic HLA, T-cell clones carrying high affinity TCRs specific for self-antigens such as CD22 can be raised. T-cell clone 9D4 demonstrated great peptide specificity, a feature also observed for other T-cell clones raised in an HLA-mismatched setting after allogeneic stem cell transplantation [26, 27]. In addition, other groups have reported comparable observations when inducing T-cell response across an HLA-mismatch *in vitro* [28–31].

T-cell clone 9D4 was selected because it demonstrated highest peptide sensitivity amongst all isolated CD22-specific T-cell clones. Clone 9D4 specifically produced cytokines upon stimulation with all 4 primary ALL samples, both ALL cell lines and normal B-cells. However, we could not establish a clear correlation between antigen expression levels and degree of cytokine production for Clone 9D4 and T-cells transduced with the CD22-specific TCR. Besides expression levels of the antigen, additional factors such as HLA molecule abundance and presence of other costimulatory and adhesion molecules on target cells may account for this difference in sensitivity. Nonetheless, primary ALL, ALL cell lines, and normal B-cells could be lysed by clone 9D4 and TCR-modified T-cells. Furthermore, clone 9D4 and TCR-engineered T-cells weakly lysed DCs and macrophages in the absence of IFN-γ production, indicating that also cells with low levels of CD22 expression can be targeted.

The expression of CD22 on non-B-cells such as DCs and macrophages poses direct limitations of CD22 as a target-antigen in immunotherapy. While permanent eradication of the B-cell lineage is permissible and hypogammaglobulinemia is clinically manageable, ablation of the dendritic cell and type I macrophage compartment is most likely not tolerable long-term. To prevent long-term depletion of the dendritic cell and macrophage compartment TCR-engineered T-cells could additionally be equipped with suicide genes to deplete the administered T-cell product from circulation after successful eradication of the malignancy [32–34]. Such a strategy could limit the potential toxicity towards undesired cell subsets, however, may also increase the chance of relapse of the malignancy once tumor-surveilling TCR-engineered T-cells have been depleted from circulation.

It should also be noted that we observed CD22 cell surface expression on DCs and macrophages which has also been reported by others [35]. Therefore, these cell subsets could be affected by on-target off-tumor toxicity of CD22-specific mAbs or CAR-engineered T-cells. No toxicity on the myeloid lineage has been described for the CD22-targeting mAb epratuzumab. However, CD22 is currently also evaluated as a target for CAR-based immunotherapies with several trials recruiting patients (www.clinicaltrial.gov). Since CAR-engineered T-cells require less antigen than mAbs [36] and can persist long term in patients, this potential off-tumor toxicity should be considered in clinical trial design.

In summary, T-cells engineered to express a CD22-specific TCR could be a treatment option in patients suffering from CD22^pos^ B-cell malignancies. However, while CD22-targeting may be feasible in antibody-based approaches, our study highlights potential limitations of CD22 as an ideal antigenic target for cellular immunotherapeutic interventions.

## MATERIALS AND METHODS

### Culture conditions and cells

All studies using human material were approved by the Leiden University Medical Center ethical review board. Peripheral blood was obtained from healthy individuals or patients after informed consent. PBMCs were isolated using Ficoll-gradient centrifugation and were cryopreserved. Primary hematopoietic cell subsets were obtained from cryopreserved PBMCs of HLA-B*07:02^pos^ healthy donors that were incubated with either anti-CD4, anti-CD8, anti-CD14, anti-CD19 or anti-CD34 magnetic microbeads (Miltenyi Biotec, Bergisch Gladbach, Germany) for 15 min at 4°C. Microbead-labeled cells were isolated on LS column (Miltenyi Biotec) according to manufacturer's protocol. Purity of isolated cells was assessed using FACS analysis and cells were only used in experiments if purity exceeded 95%. T-cells were cultured in T-cell medium consisting of IMDM (Lonza, Basel, Switzerland) supplemented with 100 IU/ml IL-2 (Proleukine; Novartis Pharma, Arnhem, The Netherlands), 5% fetal bovine serum (FBS; Gibco, Life Technologies, Carlsbad, CA) and 5% human serum. Activated T-cells were generated by stimulating CD4^+^ and CD8^+^ T-cells with irradiated (35 Gy) feeders in a 1:5 ratio in T-cell medium supplemented with 0.8 μg/ml phytohemagglutinin (PHA; Biochrom AG, Berlin, Germany) for 10 days prior to experiment. Immature and mature DCs were differentiated *in vitro* from isolated CD14^+^ cell populations. Briefly, on day zero 1×10^6^ cells/ml were seeded in IMDM supplemented with 100 ng/ml GM-CSF (Sandoz Novartis Pharma, Almere, The Netherlands), 500 IU/ml IL-4 (Schering-Plough, Kenilworth, NJ), and 10% human serum, and cultured for two days to obtain immature DCs. Mature DCs were generated by culturing immature DCs in IMDM supplemented with 100 ng/ml GM-CSF, 10 ng/ml TNFalpha (CellGenix, Freiburg, Germany), 10 ng/ml IL-1b (Bioscource Invitrogen, Camarillo, CA), 10 ng/ml IL-6 (Sandoz Novartis Pharma), 1 μg/ml PGE-2 (Sigma Aldrich, St. Louis, MO), 500 IU/ml INF-γ (Boehringer Ingelheim, Ingelheim am Rhein, Germany), and 10% human serum for an additional two days. Type I and II macrophages were *in vitro* differentiated from CD14^+^ monocytes. CD14^+^ monocytes were cultured for 8 days in IMDM containing 10% human serum in the presence of 50 ng/ml GM-CSF or 5 IU/ml CSF-1 (R&D Systems, Minneapolis, MN) to obtain type I or II macrophages, respectively. Activated CD19^+^ B-cells were generated by co-culturing CD19^+^ cells on CD40L-transduced irradiated (70 Gy) mouse-fibroblasts for 7 days in IMDM supplemented with 2 ng/ml IL-4 and 10% human serum. K562 cells expressing HLA-B7 (K562-B7) were previously described [31]. ALL cell lines were previously described [37]. Fibroblasts, keratinocytes and proximal tubular epithelial cells (PTECs) were cultured either in the absence or presence of 200 IU/ml IFN-γ for four days before cells were used in experiments. All cells were washed twice before use in experiments.

### Generation of peptide-MHC complexes

Peptide CD22_RPF_ was synthesized in-house using standard Fmoc chemisty. Recombinant HLA-B7 heavy chain and human β_2_m light chain were in-house produced in *Escherichia coli*. MHC class I refolding was performed as previously described with minor modifications [38]. MHC class I complexes were purified by gel-filtration using HPLC. pMHC-tetramers were generated by labeling biotinylated pMHC-monomers with streptavidine-coupled phycoerythrin (PE; Invitrogen, Carlsbad, CA) or allophycocyanin (APC, Invitrogen). Complexes were stored at 4°C.

### Isolation of CD22-reactive T-cell clones

T-cells binding to CD22-specific pMHC-tetramers composed of peptide CD22_RPF_ bound to HLA-B7 were isolated from PBMCs of healthy HLA-B7^neg^ individuals. PBMCs were first incubated with PE-labeled pMHC-tetramers for 1 h at 4°C. Cells were washed twice and incubated with anti-PE-microbeads (Miltenyi Biotec) for 15 min at 4°C. PE-labeled cells were isolated on an LS colomn (Miltenyi Biotec) according to manufacturer's instruction. Positively selected cells were stained with an Alexa700-labelled antibody against CD8 (Invitrogen/Calteg, Buckingham, United Kingdom) in combination with FITC-conjugated antibodies against CD4, CD14, and CD19 (BD Pharmingen, San Jose, CA). Using a AriaIII cell sorter (BD Biosciences, Franklin Lakes, NJ) and Diva Software (BD Biosciences), pMHC-tetramer^+^ CD8^+^ T-cells were single-cell sorted into round-bottom 96-well plates containing 5 × 10^4^ irradiated (35 Gy) feeders in 100 μl T-cell medium supplemented with 0.8 μg/ml PHA.

### TCR gene transfer

TCRAV and TCRBV usage of T-cell clone 9D4 was determined by reverse transcriptase (RT)-PCR and sequencing using a previously established protocol [39]. V(D)J segments of the TCR alpha and TCR beta chain were codon optimized and cloned into the modified MP71-TCR-flex retroviral backbone. To increase expression and preferential pairing of the introduced TCR alpha and beta chain, the MP71-TCR-flex vector contained codon-optimized and cysteine-modified murine TCR alpha and beta constant domains and a porcine teschovirus-derived P2A sequence to link TCR chains [40]. A complete construct was ordered from GenScript (Piscataway, NJ).

Purified CD8^+^ T-cells were activated using irradiated autologous PBMCs and PHA. On day 2 following stimulation, retroviral supernatant containing either TCR-9D4 or an empty backbone (Mock) was loaded on 24-well nontissue culture–treated plates that had been coated with 30 mg/mL retronectine (Takara, Shiga, Japan) and blocked with 2% human serum albumin (Sanquin Reagents, Amsterdam, The Netherlands). Viral supernatant was spun down at 2000 *g* for 20 minutes at 4°C before activated T-cells were added to retroviral supernatant and incubated at 37°C for 18 hours. 7 days after stimulation, high-purity TCR-transduced T-cells were obtained by MACS isolation based on the expression of the transduced TCR or marker gene nerve growth factor-receptor (NGF-R also known as CD271). Transduced T-cells were incubated with an APC-labelled antibody against the murine constant TCR domain (BD Pharmingen) or NGF-R (Sanbio, Uden, The Netherlands) for 15 min at 4°C and washed twice. Following incubation with anti-APC microbeads (Miltenyi Biotec) for 15 min at 4°C, TCR-transduced T-cells were isolated on a LS column following manufacturer's instructions.

### FACS analysis

FACS acquisition was performed on a LSRII (BD Biosciences) or a FACS Calibur (BD Biosciences) and was analyzed using Diva Software (BD Biosciences) or FlowJo Software (TreeStar, Ashland, OR). Isolated T-cell clones were analyzed for binding to specific pMHC-tetramers and CD8 expression by staining with PE-labelled pMHC-tetramers, and an Alexa700-conjugated antibody against CD8 (Invitrogen/Calteg) combined with FITC-labelled antibodies against CD4, CD14, and CD19 (BD Pharmingen). 10,000 cells of a T-cell clone were first incubated with 2 μg/ml pMHC-tetramers for 15 min at 37°C before antibodies were added and incubated for an additional 15 min at 4°C. Similarly, 25,000 TCR-transduced or mock-transduced T-cells were incubated with 2 μg/ml pMHC-tetramers for 15 min at 37°C before antibodies against CD8, CD4, NGF-R or murine TCR beta constant domain were added and incubated at 4°C for 15 min. PBMCs, purified hematopoietic cell subsets or activated cells were stained with antibodies against CD3, CD4, CD14, CD19, CD34 (BD Pharmingen), CD22 (clone S-HCL-1, BD Pharmingen) or an isotype control for 4°C for 15 min.

### Functional analysis

K562 cells were peptide-pulsed at titrated peptide concentrations for 30 min at 37°C. Responder T-cells and peptide-pulsed or unloaded stimulator cells were co-incubated at various responder-to-stimulator ratios. After 18 hours coincubation, supernatants were harvested and IFN-γ or GM-CSF production was measured by enzyme-linked immunosorbent assay (ELISA, Sanquin Reagents or R&D Systems, respectively).

### FACS-based cytotoxicity assay

Adapted from Jedema *et al.* [41], 10,000 PKH26GL-labelled (Sigma-Aldrich) target cells were co-incubated with T-cells at various effector-to-target ratios in 50 μl T-cell medium for 20 hours. After coincubation, cells were stained with Sytox Blue dead cell stain (Invitrogen/Caltag) in a final concentration of 1 μM for 5 min. 10 μl Flow-count fluorospheres (Beckman Coulter, Brea, CA) were added and samples were analyzed using FACS. For each sample, 3,300 Flow-count fluorospheres were acquired and the percent surviving cells was calculated as follows: [(PKH26GL-labelled targets in the presence of effector cells)/(PKH26GL-labelled targets in absence of effector cells)] × 100%.

### Quantitative real-time PCR of CD22

From isolated cell subsets or cultured cells, total RNA was isolated using the RNAqueous Micro-Kit and Small Scale Kit (Ambion, Life Technologies) for a maximum of 0.5 × 10^6^ and 10 × 10^6^ cells, respectively, following manufacturer's instructions. Total RNA was converted to cDNA using M-MLV reverse transcriptase (Invitrogen). *CD22* expression was measured on the Roche Lightcycler 480 (Roche) using Fast Start TaqDNA Polymerase (Roche) and EvaGreen (Biotium, Hayward, CA) with forward primer 5′-CGACGTTGGAAGAGGACACA-3′ and reverse primer 5′-GGGGGCCCTTCTAACCTTTT-3′.

## SUPPLEMENTARY MATERIALS FIGURES AND TABLES


