# Supplementary material for: A CD22-reactive TCR from the T-cell allorepertoire for the treatment of acute lymphoblastic leukemia by TCR gene transfer
Source: Oncotarget. 2016 Sep 26;7(44):71536–47. doi: 10.18632/oncotarget.12247 (PMC5342099; doi:10.18632/oncotarget.12247)
Supplement: Supplementary file 1 [file oncotarget-07-71536-s001.pdf]

A CD22-reactive TCR from the T-cell allorepertoire for the treatment of acute lymphoblastic leukemia by TCR gene transfer

Supplementary Materials

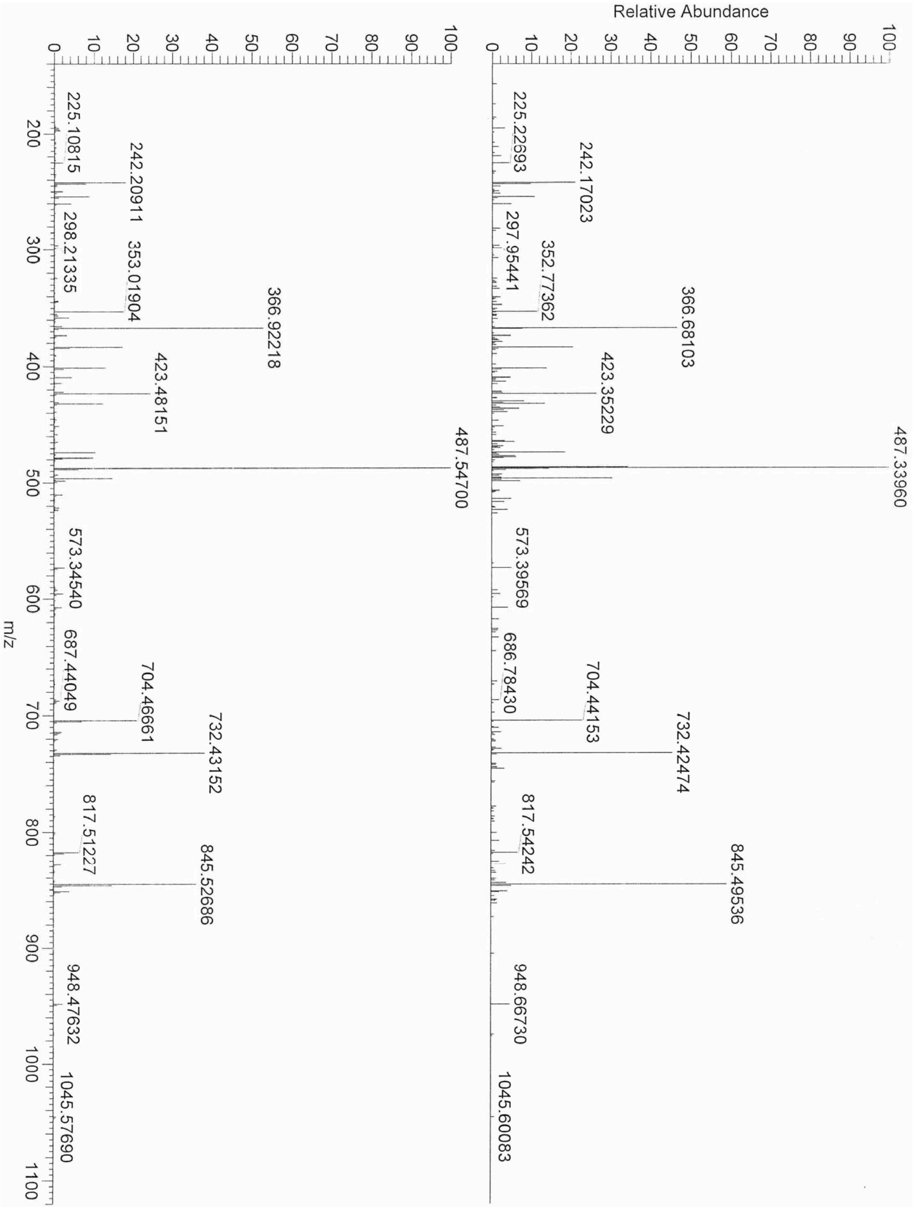

Supplementary Figure S1: Matching tandem mass spectra of synthesized and eluted CD22-derived peptide RPFPPHIQL ($CD22_{RPF}$). Shown are tandem mass spectra of the eluted (top) and synthesized (bottom) peptide.

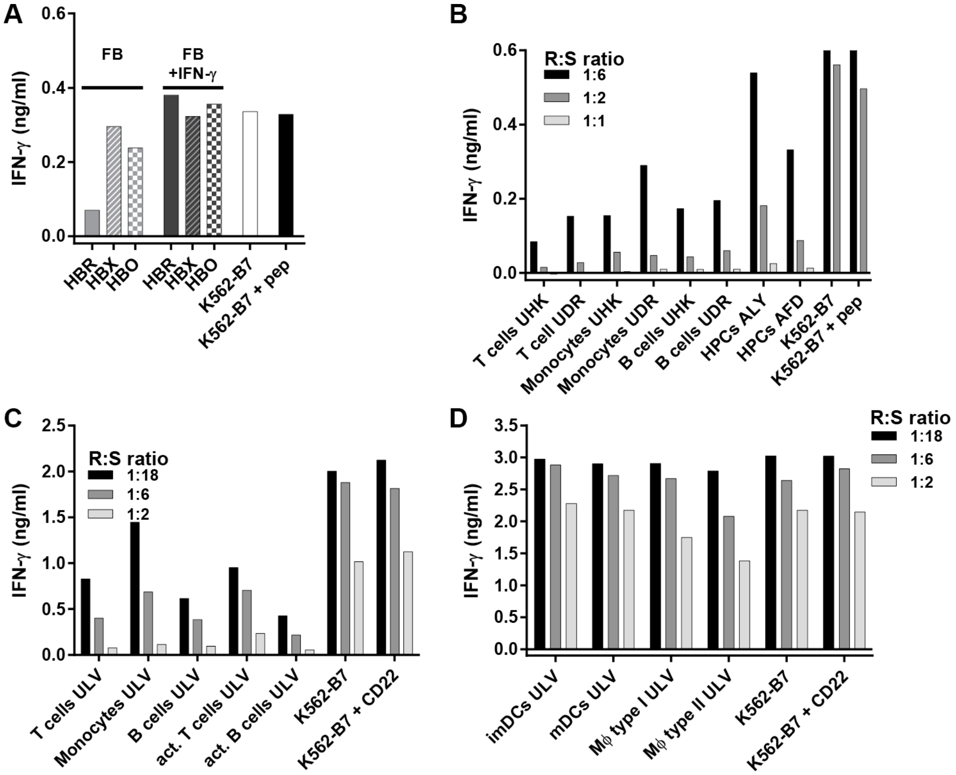

Supplementary Figure S2: Recognition of stimulator cells by control T-cell clone CTZ. Control T-cell clone CTZ recognizing a ubiquitously expressed antigen presented in HLA-B7 was used to test the stimulatory capacity of used cell samples. (**A**–**C**) The same stimulator cells as in Figure 3A–3C, respectively, were cocultured with clone CTZ. (**D**) The stimulatory capacity of cells used in Figure 4A was tested using clone CTZ.

Supplementary Table S1: HLA typing of B-LCL panel used in this study (Adapted from van Loenen *et al.* [25])

	HLA class I			HLA class II		
**B-LCL**	**A**	**B**	**C**	**DR**	**DQ**	**DP**
NGZ	11, 24	53, 61	2	1, 11	5, 7	1*0201, 1*0301
LMB	29	44, 51	14, 16	7, 8	2, 4	1*0401, 1*110101
MWX	1, 34	15, 35	4, 12	1, 15	5, 6	1*0601, 1*1301
ABV	3, 29	7, 44	7	7, 14	5, 2	1*0401, 1*1101
UKL*	1, 30	8, 13	6, 7	4, 17	2, 7	1*0401
AKB	1, 2	37, 39	6, 7	1, 10	5	2*0102, 1*0401
RBB*	2, 26	8, 27	1, 7	17, 52	2	1*0401
IZA	2, 24	8, 60	3, 7	13, 17	2, 6	1*0401, 1*1401
NGI	11, 24	8, 39	7	17, 8	2, 4	1*0101, 1*1401
AHT	24, 25	55, 62	3	8, 15	5, 6	1*0401
MHX	1, 0205	18, 50	6, 7	7, 9	2, 9	1*0201, 1*0301
CVV	11, 31	57, 62	3, 6	4, 7	7, 9	1*0201, 1*0401
UCE*	3, 11	7, 27	2, 7	11, 14	5, 7	1*0201, 1*1601
MBX	1	8, 63	7	12, 17	2, 7	1*0101, 1*0401
EBM	23	64	8	4	8	1*0201
FAQ	23, 68	14, 38	8, 12	13	7, 6	1*0201
LSR	32, 68	35, 52	12	15, 16	5, 6	1*0401, 1*1401
GGT	26, 31	14, 49	7, 8	1, 7	2, 5	1*0402, 1*110101

*B-LCL not used in previous study and added in this panel.
